# Integrating unified medical language system and association mining techniques into relevance feedback for biomedical literature search

**DOI:** 10.1186/s12859-016-1129-z

**Published:** 2016-07-19

**Authors:** Yanqing Ji, Hao Ying, John Tran, Peter Dews, R. Michael Massanari

**Affiliations:** 1 Department of Electrical and Computer Engineering, Gonzaga University, Spokane, WA USA; 2 Department of Electrical and Computer Engineering, Wayne State University, Detroit, MI USA; 3 Frontier Behavioral Health, Spokane, WA USA; 4 Department of Medicine, St. Mary Mercy Hospital, Livonia, MI USA; 5 Research for The Critical Junctures Institute, Bellingham, WA USA

**Keywords:** Biomedical literature search, Relevance feedback, Association mining, UMLS

## Abstract

**Background:**

Finding highly relevant articles from biomedical databases is challenging not only because it is often difficult to accurately express a user’s underlying intention through keywords but also because a keyword-based query normally returns a long list of hits with many citations being unwanted by the user. This paper proposes a novel biomedical literature search system, called BiomedSearch, which supports complex queries and relevance feedback.

**Methods:**

The system employed association mining techniques to build a *k*-profile representing a user’s relevance feedback. More specifically, we developed a *weighted interest* measure and an association mining algorithm to find the strength of association between a query and each concept in the article(s) selected by the user as feedback. The top concepts were utilized to form a *k*-profile used for the next-round search. BiomedSearch relies on Unified Medical Language System (UMLS) knowledge sources to map text files to standard biomedical concepts. It was designed to support queries with any levels of complexity.

**Results:**

A prototype of BiomedSearch software was made and it was preliminarily evaluated using the Genomics data from TREC (Text Retrieval Conference) 2006 Genomics Track. Initial experiment results indicated that BiomedSearch increased the mean average precision (MAP) for a set of queries.

**Conclusions:**

With UMLS and association mining techniques, BiomedSearch can effectively utilize users’ relevance feedback to improve the performance of biomedical literature search.

## Background

A large volume of clinical and basic research articles are published in the biomedical field each year, which are available online. The most influential biomedical database is PubMed [1] developed and maintained by the National Center for Biotechnology Information of the Library of Medicine. PubMed includes more than 24 million citations and approximately 10,000 citations are added to the database every week. These articles provide an important source of information that not only enables biologists to discover in-depth knowledge about various biological systems, but also helps healthcare professionals do evidence-based medicine in clinical settings [2, 3]. However, finding highly relevant articles from biomedical databases is challenging due to the huge number of articles and users’ difficulty in accurately expressing their information needs.

PubMed supports keyword and constraint queries. However, a keyword query normally returns a long list of hits. And, many citations are not what the user is looking for even though they meet the keyword search criteria. For example, the keyword “Parkinson’s disease” retrieves more than seventy thousand articles. Adding a couple of constraints could narrow down the results but the returned list is still likely too long for users to review each hit. Furthermore, the quality of the query results is poor when users only vaguely know what they need and cannot provide precise keywords.

To shorten the returned results and improve the query quality, researchers have studied various querying strategies. For example, Murphy et al. attempted to use controlled vocabulary and key terms to formulate appropriate queries [4]. Sneiderman and his colleagues explored how knowledge-based approaches could facilitate finding practical clinical advice in the biomedical literature [5]. Instead of studying different querying methodologies, a couple of researchers tried to utilize clustering techniques or biomedical ontologies to re-organize the presentation of the returned results to users [6, 7]. Some other researchers have investigated how to employ citation information to compute the importance of articles and apply it to rank the results [8, 9]. This ranking may not conform to users’ query intentions due to the fact that, even with the same keyword query, users’ specific information needs are typically widely varied [10]. Machine learning techniques have also been applied to search relevant articles by ranking articles according to a learned relevance function [11, 12]. One limitation of these techniques is that a large number of training articles must be provided in order to achieve a reasonable learning accuracy.

Relevance feedback technique represents an established technique in information retrieval to improve retrieval performance [13]. It has been applied to biomedical literature search [10, 14]. This technique utilizes users’ feedback, implicitly or explicitly, on previous search results to generate new search results that are supposedly more closely related to users’ specific information needs. The use of this technique in biomedical literature search is still limited. States et al. proposed an implicit relevance feedback approach [14] that automatically save information on citations a user has viewed during search and browsing, and uses this information to construct a statistical profile representing the user’s choices. This profile is then employed to rank future searches. Yu et al. developed a multi-level relevance system, called RefMed, for PubMed [10]. Once a user’s feedback is received, the system induces a relevance function from the feedback using a learning method called RankSVM. This function is then used to rank the results. Like PubMed, both relevance feedback systems support keyword queries for initial search. Thus, the effectiveness of these systems partially depends on users’ ability in selecting proper keywords. If keywords are not properly chosen, the top returned results may not include any relevant articles, which makes relevance feedback systems not work. On the other hand, these systems do not support complex topic or question queries where each query may contain punctuation, stop words, etc. The reason is that these queries may return nothing for initial search, which also makes relevance feedback systems not work.

In this paper, we propose a novel relevance feedback system, called BiomedSearch, for biomedical literature search which is designed to support complex topic queries where each topic can be one or more keywords, a question with stop words, or even a paragraph describing a topic of interest. The system conducts the search process using UMLS knowledge sources, text mining techniques, relevance feedback approach, and association mining techniques. Specifically, BiomedSearch has the following key features:

- BiomedSearch is supported by UMLS (Unified Medical Language System) knowledge sources. Both search topics and articles are converted to standard biomedical concepts using UMLS Metathesaurus, a biomedical vocabulary and standard database. The matching between a topic and each article is done through these standard concepts instead of ad-hoc keywords.
- BiomedSearch supports topic queries with any levels of complexity. Each topic can include any number of keywords, questions, or sentences. Most keyword-based search engines do not support complex topic search. For example, if a question “How do Cathepsin D (CTSD) and apolipoprotein E (ApoE) interactions contribute to Alzheimer’s disease?” is searched in PubMed, nothing is returned.
- Association mining techniques are integrated into the relevance feedback approach for next-round article retrieval. Specifically, once a user “pushes the feedback,” association mining techniques are used to compute the strength of association between the search topic and each biomedical concept in the selected article(s). We propose a *weighted interest* measure and an association mining algorithm to evaluate the strength of associations. The top *k* concepts form a profile which represents the user’s intention. This profile is then matched with each article and places those articles that the user is most like to view at the top of the next returned list. More details about the application of association mining techniques will be discussed in Section III. To the best of our knowledge, our work is the first attempt to integrate association mining into relevance feedback for biomedical literature search.
- The relevance feedback mechanism used by BiomedSearch requires minimum user interactions. Users only need to provide whether an article is relevant or not without further details. In addition, the users can select any number of relevant articles.

## Background on UMLS and association mining

### UMLS

The UMLS is a set of files and software that brings together many health and biomedical vocabularies and standards that can be used to enhance or develop biomedical and health-related applications, such as electronic health records, classification tools, dictionaries and language translators. It also enables interoperability between computer systems. The UMLS contains three tools which are called knowledge sources: Metathesaurus, Semantic Network, and SPECIALIST Lexicon and Lexical Tools. The Semantic Network and Lexical Tools to were used to produce the Metathesaurus. However, each tool can be accessed separately or in any combination according to users’ needs. In this study, the Metathesaurus were used to convert free text to standard biomedical concepts.

The UMLS Metathesaurus comprises over 1 million health and biomedical concepts from over 100 controlled vocabularies such as International Classification of Diseases version 10 (ICD-10), Medical Subject Headings (MeSH), etc. Each concept has a unique identification (ID) as well as specific attributes defining its meaning. The UMLS Metathesaurus has been applied to several biomedical information retrieval fields such as classification [15, 16], re-organization of search results [17], matching patient records to biomedical articles [18], relation extraction [19], semantic similarity [20], and medical question answering [21].

### Association mining

Association mining intends to discover association rules in the form of *X* → *Y* from large datasets, where *X* and *Y* are two disjoined itemsets, i.e., *X* ∩ *Y* = Ø [22]. An association rule indicates that the presence of *X* implies the presence of *Y*. Both *X* and *Y* can have one or more items. Association mining was first proposed to discover regularities between products in large-scale transaction data from supermarkets [22]. For example, the rule {*cheese, milk*} → {*eggs*} found in the sales data of a supermarket would indicate that if customers buy cheese and milk together, they are likely to also buy eggs. Such information can be utilized as the basis for decisions about marketing activities such as promotional pricing or product placements.

The strength of an association rule is assessed by various interestingness measures such as *confidence* [22], *IS* [23], Klosgen’s measure [24], *interest* [25], and so forth. The definitions of these measures are typically based on the frequency counts related to both *X* and *Y* in a dataset. Many researchers have applied various measures and algorithms to mine different types of data, especially in the medical domain where finding the potential associated factors for particular medical conditions is a fundamental objective [26–33]. For instance, Jin et al. attempted to mine unexpected associations with applications in signaling potential adverse drug reactions caused by a single drug using administrative health databases [27]. They tried to discover associations between two events *X* and *Y* where *Y* occurs unexpectedly within a period *T* after *X*. Noren et al. proposed another association mining method which contrasts the observed-to-expected ratio in a time period after *X* to the observed-to-expected ratio in a control period before *X* [26]. Concaro et al. extended traditional temporal association mining by handling both point-like events and interval-like events (e.g., drug consumption) [29].

### Methods

**Fig. 1:**
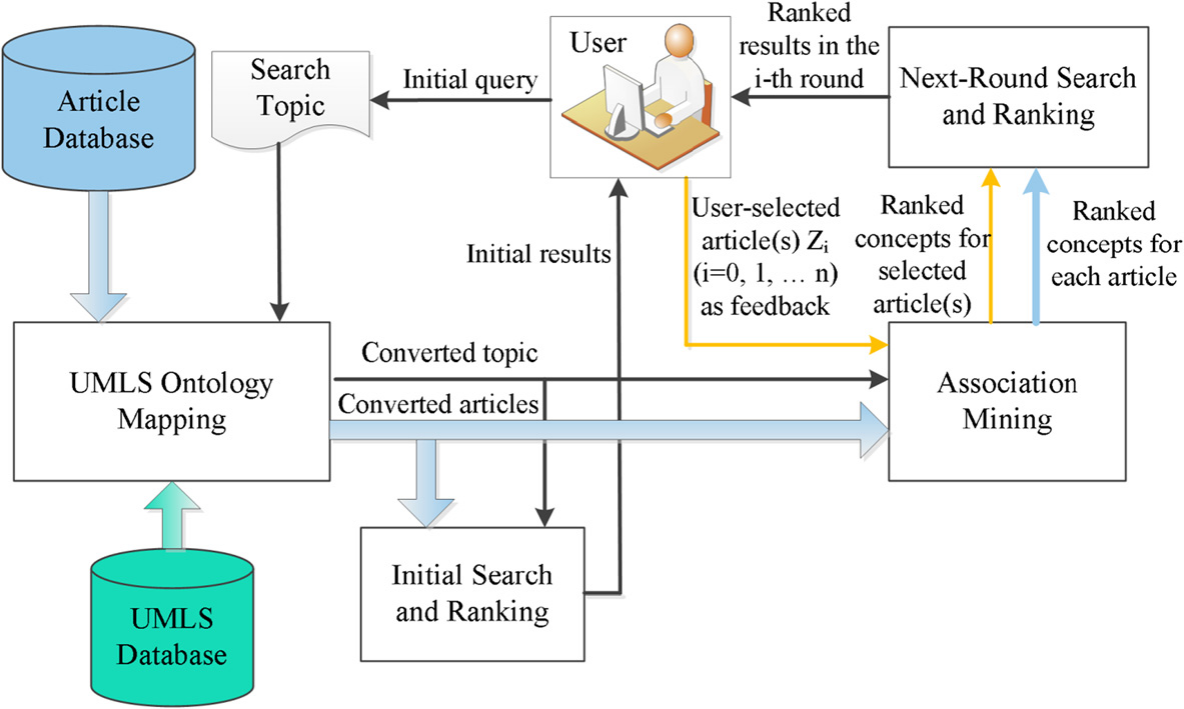
BiomedSearch System Architecture

Figure 1 presents the BiomedSearch system architecture. A user can trigger the system by entering a topic of interest. The topic as well as all the articles is converted to standard biomedical concepts. The concepts in the topic are used to match those in each article in order to return an initial ranked list for the user. The user reviews the initial results and selects one or more articles as relevance feedback. After that, association mining techniques are used to rank the concepts in the selected article(s) according to their strength of association with the search topic. The top *k* concepts are selected to represent the user’s intention. The same process is utilized to find the top *k* concepts in each of the articles. All the articles are ranked based on the similarity between the top *k* concepts from each article and those from the selected article(s). The user can do multi-round relevance feedback until he/she finds the desirable articles. The details of each component in Fig. 1 are described below.

### UMLS ontology mapping

In BiomedSearch, the whole search process is conducted using standard biomedical concepts instead of ad-hoc keywords or terms. In the context of this study, a biomedical concept refers to a standard biomedically meaningful term with a unique identification defined in the UMLS Metathesaurus.

We assume that all articles are stored in a database. If the articles are not text files (e.g., pdf, html), they need to be converted to text files. In order to map articles to biomedical concepts, the text files are sent to UMLS servers one by one through Java-based APIs provided by UMLS. The UMLS servers are maintained by National Library of Medicine (NLM). These servers hold the Metathesaurus and a set of lexical tools. Once a text file is received by the servers, it is broken down into sentences, each of which is further broken into phrases. Each phrase is mapped to one or more standard concepts in Metathesaurus by a lexical tool called MetaMap [34]. The servers generate a MetaMap file containing each phrase and its matched concepts and return it to users’ local computer. Note that each phrase may be mapped to multiple concepts, each of which is associated with a score. The higher the score, the closer the phrase matches the concept.

**Fig. 2:**
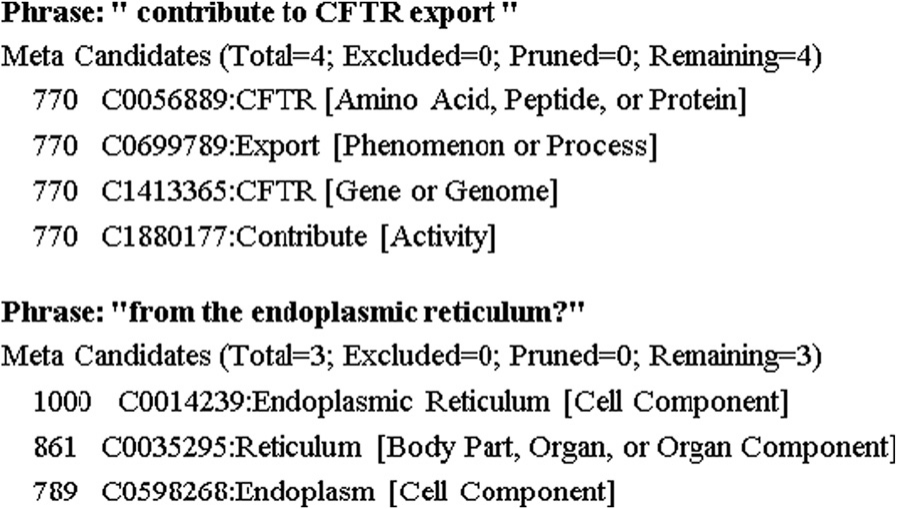
Example Phrases and Their Matched Concepts in a MetaMap File

Figure 2 presents two example phrases and their matched concepts in a MetaMap file. The number at the beginning of each line is the matching score. The code started with ‘C’ represents the unique concept identification (ID) number for the matched biomedical concept shown next. The term within the bracket at the end of each line is the sematic type of the biomedical concept.

In UMLS, semantic types represent a set of broad subject categories that provide a consistent categorization of all concepts defined in the UMLS Metathesaurus.

After the MetaMap file for each article is obtained, the mapped concept IDs for each phrase in a MetaMap file are extracted and saved in a new file. If a phrase is mapped to multiple concepts, those concept IDs whose scores are larger than a limit are retrieved. In this study, the score limit is set 500 by the biomedical professionals in our project team. Given the two example phrases in Fig. 2, seven concept IDs are extracted, one for each mapped concept. In addition, if a phrase appears multiple times in an article, its mapped concept IDs will be recorded multiple times in the new file. With the same procedure, users’ queries can also be converted to concept IDs which represent users’ information needs. The following matching and processing will only deal with these concept IDs.

### Initial search and ranking

As we mentioned in Introduction, the initial search is also important and must be effective. If the initial top results do not include any relevant articles, a user has to review more articles deep in the returned list. The number of articles that will be reviewed by a user depends on the user’s patience and available time. In this study, we use accumulative term frequency-inverse document frequency (TF-IDF) to rank the articles for the initial search. TF-IDF is an established weighting scheme in information retrieval and text mining [35]. It overweights a term by its frequency in the document and underweights it by the log of how common it is in a collection of documents. It essentially makes the TF-IDF value higher for a term that has high frequency in a document but is less likely contained by the other documents in a collection. In this context, a term is actually a concept ID and a document refers to a biomedical article. To be consistent with the notation of TF-IDF, we use the term “document” to represent an article in the following discussions.

Let *D* = {*d*_1_, *d*_2_, …, *d*_1_, … *d*_*m*_} be a set of documents. Let *C* = {*c*_1_, *c*_2_, …, *c*_*j*_, … *c*_*n*_} be a set of unique biomedical concepts contained by a document *d*_*i*_. Term frequency (TF) measures how often a term appears in a document. Terms that appear in a document more times are more likely to be important within the document. The term frequency for a concept *c*_*j*_ in a document *d*_*i*_ is defined as the frequency of *c*_*j*_ in *d*_*i*_ divided by the total number of concepts in *d*_*i*_. That is,

1 $$ T{F}_{c_j}^{d_i}=\frac{f_{c_j}^{d_i}}{{\displaystyle {\sum}_{j=1}^n}{f}_{c_j}^{d_i}} $$

where ${f}_{c_j}^{d_i}$ represents the frequency of a concept *c*_*j*_ in *d*_*i*_. The inverse document frequency (IDF) examines the general importance of a term in a set of documents *D*. It is defined as

2 $$ ID{F}_{c_j}^D= log\frac{\left|D\right|}{D{F}_{c_j}} $$

where |*D*| represents the total number of documents in *D*, and *DF*_*cj*_ is the total number documents that contain the concept *c*_*j*_. The TF-IDF weight of a concept *c*_*j*_ in *d*_*i*_ is defined as its TF multiplied by IDF. That is,

3 $$ TF- ID{F}_{c_j}^{d_i}=T{F}_{c_j}^{d_i}* ID{F}_{c_j}^D=\frac{f_{c_j}^{d_i}}{{\displaystyle {\sum}_{j=1}^n}{f}_{c_j}^{d_i}}\ast log\frac{\left|D\right|}{D{F}_{c_j}} $$

Let *Q* = {*c*_1_, *c*_2_, …, *c*_*k*_ …, *c*_*l*_} be a query that typically contains a much smaller set of concepts. We use accumulative TF-IDF weights of all the concepts in *Q* to rank all the documents in *D*. That is, for each document, we first compute the TF-IDF weight of each concept in *Q* and then sum up these weights. We define accumulative TF-IDF, named A-TF-IDF, for a document *d*_*i*_ relative to a query *Q* as below:

4 $$ A-TF- ID{F}_Q^{d_i}={\displaystyle \sum_{k=1}^lTF- ID{F}_{c_k}^{d_i}}={\displaystyle \sum_{k=1}^lT{F}_{c_k}^{d_i}* ID{F}_{c_k}^D\kern2em ={\displaystyle {\sum}_{k=1}^l\left(\frac{f_{c_k}^{d_i}}{{\displaystyle {\sum}_{j=1}^n}{f}_{c_j}^{d_i}}*\  log\frac{\left|D\right|}{D{F}_{c_k}}\right)}} $$

After the A-TF-IDF is computed for each document, the documents are ranked according to their A-TF-IDF values. The document with a higher A-TF-IDF value will be ranked higher. The ranked list is then returned as initial results to users. The users can review the top documents and select one or more relevant documents as feedback for further search.

### Association mining

Once BiomedSearch receives the user-selected document(s) *Z* as feedback, association mining techniques are employed to find the strength of association between the query *Q* and each unique concept in *Z*. The concepts in *Z* are ranked according to their strength of association with *Q*. The top *k* concepts are then selected to form a profile that represents the user’s query interest and is used for next-round search.

In this study, we extend the *interest* measure and define a *weighted interest* measure. The original *interest* measure, *I*, is defined as

5 $$ I=\frac{N{f}_{XY}}{f_X*{f}_Y} $$

where *f*_*x*_ and *f*_*y*_ represent the number of transactions/records that contain *X* and *Y*, respectively. N is the total number transactions in the dataset and *f*_*XY*_ is the total number of transactions that contain both *X* and *Y*. The *I* measure is inspired by the statistical independence theory. That is, If *X* and *Y* are statistically independent, then *P* (*X*, *Y*) = *P* (*Y*) × *P* (*Y*). The above definition can be transformed to the following format:

6 $$ I=\frac{f_{XY}/N}{\left({f}_X/N\right)\ast \left({f}_Y/N\right)} $$

One can see that *f*_*XY*_/*N* is an estimate for the joint probability *P* (*X*, *Y*), while *f*_*X*_/*N* and *f*_*Y*_/*N* are the estimates for *P*(*X*) and *P*(*Y*), respectively. Therefore, the *I* measure compares the frequency of a pattern against a baseline frequency obtained under the statistical independence assumption. The measure indicates an association if its value is larger than 1.

In this study, a query *Q* and each concept in the user-selected document(s) *Z* forms an association rule, i.e., *Q →* {*c*_*j*_}, where *c*_*j*_ represents a concept in *Z*. The total number of association rules is equal to the number of unique concepts covered by *Z*. To apply association mining techniques, we split *Z* into sentences where each sentence is analogous to a transaction and contains a list of concepts. This split is reasonable since concepts that appear in the same sentence generally have stronger relationships. However, since *Q* may include multiple concepts, the chance that all these concepts appear in the same sentence is low. This would cause the frequency of *Q* (i.e., *f*_*Q*_) to be vey low or even zero. To solve this problem, we propose a *weighted interest* measure, called *I*^*w*^, to support partial count when only part of *Q* is contained by a sentence. The partial count of *Q* in a sentence *s*_*i*_ is defined as the number of concepts contained by the sentence divided by the total number of concepts in *Q*. That is,

7 $$ CNT{(Q)}_{S_i}=\frac{\left|\left\{c\Big|c\in Q,\ c\in {s}_i\right\}\ \right|}{\left|Q\right|} $$

where | | represents the total number of elements in a set. With this definition, the count of *Q* is not binary (i.e., 0 or 1) any more. It can be any value between 0 and 1. We define weighted frequency of *Q* below:

8 $$ {f}_Q^w={\displaystyle {\sum}_{i=1}^{\left|Z\right|}CNT{(Q)}_{s_i}}\kern1em ={\displaystyle {\sum}_{i=1}^{\left|Z\right|}\frac{\left|\left\{c\Big|c\in Q,\ c\in {s}_i\right\}\ \right|}{\left|Q\right|}} $$

where |*Z*| represents the total number of sentences in *Z*.

The count of *c*_*j*_ in a sentence *s*_*i*_ is still binary. If the sentence contains *c*_*j*_, the count of *c*_*j*_ is 1. Otherwise, it is 0. i.e.,

9 $$ CNT{\left({c}_j\right)}_{s_i}=\left\{\begin{array}{c}\hfill 1,\kern2em  if\ {c}_j\in {s}_i\hfill \\ {}\hfill 0,\kern1em  otherwise\hfill \end{array}\right. $$

The frequency of *c*_*j*_ in Z is the sum of each count:

10 $$ {f}_{c_j}={\displaystyle {\sum}_{i=1}^{\left|Z\right|}CNT{\left({c}_j\right)}_{s_i}} $$

The partial count of *Q* ∪ *c*_*j*_ in a sentence is defined as

11 $$ CNT{\left(Q\cup {c}_j\right)}_{s_i}=CNT{(Q)}_{s_i}*CNT{\left({c}_j\right)}_{s_i} $$

Since $CNT{\left({c}_j\right)}_{s_i}$ is either 1 or 0, $CNT{\left(Q\cup {c}_j\right)}_{s_i}$ is equal to $CNT{(Q)}_{s_i}$ if *c*_*j*_ ∈ *s*_*i*_. Otherwise, $CNT{\left(Q\cup {c}_j\right)}_{s_i}$ is 0. The weighted frequency of *Q* ∪ *c*_*j*_ in Z is defined below:

12 $$ {f}_{Q{c}_j}^w{\displaystyle {\sum}_{i=1}^{\left|Z\right|}CNT{\left(Q\cup {c}_j\right)}_{s_i}} $$

Given the above definitions, the *weighted interest* measure, relative to *c*_*j*_, is defined as

13 $$ {I}_{c_j}^w=\frac{N{f}_{Q{c}_j}^w}{f_Q^w*{f}_{c_j}} $$

Using this measure, we can calculate the strength of association between a query *Q* and each concept in the user-selected document(s) *Z*. Note that these calculations are same, no matter whether the user selects one or more articles as feedback. After the calculations are completed, all the concepts in *Z* can be ranked according to their *I*^*w*^ values.

Next, we demonstrate the use of this measure through a simple document that contains only five sentences as shown in Table 1. We assume that each integer is a concept ID that represents a unique concept. One can see that this document contains six unique IDs. Since a query *Q* can form an association rule with each ID, six rules will be formed. For example, *Q* can be paired with {1} and form an association rule *Q* → {1}. If we assume *Q* = {3, 2, 6}, the rule can be represented as {3, 2, 6} → {1}. Given the example document and *Q*, we can use equations (7), (9), (11) to compute various counts related to each sentence. For example, with *s*_*1*_, $CNT{(Q)}_{s_1}=1/3$ since *s*_*1*_ only contains one concept in *Q*. Similarly, $CNT{\left(\left\{1\right\}\right)}_{s_1}=1$ using (9). Given these two counts, $CNT{\left(Q\cup \left\{1\right\}\right)}_{s_1}=CNT{(Q)}_{s_1}*CNT{\left(\left\{1\right\}\right)}_{s_1}=1/3$. We can compute these counts for other sentences in the same way. Table 2 lists the different counts for each sentence. Note that the sum of each count in a column is the corresponding frequency, i.e., *f*_*Q*_^*w*^, *f*_{1}_, and *f*_*Q*{1}_^*w*^. Given these frequency values, the *weighted interest* measure for the association rule {3, 2, 6} → {1} can be computed using (13). That is,

**Table 1:** Example article selected as feedback by a user

Sentence	Concept IDs
S_1_	1, 3, 4, 3, 5
S_2_	4, 5, 5, 1
S_3_	3, 5, 1, 3, 1, 6
S_4_	1, 5, 4, 4, 1
S_5_	5, 2, 4, 6, 2

**Table 2:** Counts and frequencies given the example article and *Q*

Sentence	*CNT* (*Q*)*S*_*i*_	*CNT* ({1})*S*_*i*_	*CNT*(*Q* ∪ {1})*S*_*i*_
S_1_	1/3	1	1/3
S_2_	0	1	0
S_3_	2/3	1	2/3
S_4_	0	1	0
S_5_	2/3	0	0
Sum	5/3 (*f*_*Q*_^*W*^)	4 (*f*_{1}_)	1 (*f*_*Q*{1}_^*W*^)

$$ {I}_{\left\{1\right\}}^w=\frac{N{f}_{Q\left\{1\right\}}^w}{f_Q^w*{f}_{\left\{1\right\}}}=\frac{5*1}{5/3*4}=0.75 $$

Similarly, the ${I}_{c_j}^w$ values can be computed for other concepts in the example document.

Given a query *Q* and the user-selected document(s) *Z*, we developed an association mining algorithm in order to find each association rule *Q* → *c*_*j*_ and its ${I}_{c_j}^w$ value as shown in Algorithm 1. The function *getAllSentences(Z)* reads all the sentences from *Z*, where each sentence contains a list of concept IDs. The function *getAllUniqueConcepts(Z)* obtains all distinctive concept IDs from *Z*. For each concept *c*_*j*_ ∈ *C*, the three frequencies *f*_*Q*_^*w*^, *f*_*cj*_ and *f*_*Qcj*_^*w*^ are first initialized to zeros. The inner loop (line 5–15) then iterates each sentence, computes $CNT{(Q)}_{s_i}$, $CNT{\left({c}_j\right)}_{s_i}$, and $CNT{\left(Q\cup {c}_j\right)}_{s_i}$, and adds the counts to their corresponding frequencies, respectively. The function *getPartialCnt (s*_*i*_*Q)* actually implements (7) in order to get partial counts given *Q* and a sentence *s*_*i*_. After the inner loop, *f*_*Q*_^*w*^, *f*_*cj*_ and *f*_*Qcj*_^*w*^ are obtained and then used to compute ${I}_{c_j}^w$.

After the ${I}_{c_j}^w$ value for each concept is obtained, all the concepts are ranked according to their ${I}_{c_j}^w$ values. The top *k* concepts form a *k*-profile, *P*_*k*_^*Z*^, to represent the user’s intention, which is obtained from the user-selected document(s) *Z*. We use the same procedure to obtain a *k*-profile for each document in the whole collection of documents *D*. The *k*-profile for a document *d*_*i*_, called ${P}_k^{d_i}$, represents the relevance of the document to the query *Q*.

### Next-round search and ranking

Since the user-selected document(s) *Z* generally contains more complete information about the user’s intention than *Q*, *P*_*k*_^*Z*^ is used for the next-round search and ranking. Specifically, the similarity between *P*_*k*_^*Z*^ and ${P}_k^{d_i}$ for each document is computed and the document with a higher similarity value will be ranked higher.

To find the similarity between two rank lists *P*_*k*_^*Z*^ and ${P}_k^{d_i}$, a rank-based similarity measure is needed. After examining various similarity measures in the literature, we finally choose a rank similarity measure called *rank biased overlap* (*RBO*) [36] because it has a couple of important features suitable for this study. First, it is top-weighted, placing greater emphasis on concepts ranked higher, and lesser emphasis on concepts ranked lower. Second, *RBO* can handle incomplete rankings, where a concept appearing in one rank list may not appear in the other. Third, the measure does not assign a cutoff depth *k* and the similarity results are consistent for whatever depth is available.

Let *P*_*depth*_^*Z*^ and ${P}_{depth}^{d_i}$ represent profiles derived from *Z* and *d*_*i*_, respectively, at a depth between 1 and *k*. That is, these two lists include the top *depth* concepts from *P*_*k*_^*Z*^ and ${P}_k^{d_i}$, respectively. In the context of this study, *RBO* is defined as

14 $$ RB{O}_{Z,{d}_i}=\left(1-\varphi \right){\displaystyle {\sum}_{depth=1}^k{\varphi}^{depth-1}\frac{\left|{P}_{depth}^Z\cap {P}_{depth}^{d_i}\right|}{depth}} $$

where $\left|{P}_{depth}^Z\cap {P}_{depth}^{d_i}\right|$ is the size of the *overlap* of lists *P*_*depth*_^*Z*^ and ${P}_{depth}^{d_i}$, while $\left|{P}_{depth}^Z\cap {P}_{depth}^{d_i}\right|/ depth$ represents the *agreement* of the two lists. The parameter 0 < *φ* < 1 determines how deep the decline in weights: the smaller *φ*, the more top-weighted is the measure. 1 − *φ* is a normalization factor that maps the value of *RBO* into the range [0:1]. One can see that *RBO* essentially computes a weighted average of agreement across depths, where the weights decay geometrically with depth. In this study, we set φ =0.9, a typical choice.

To demonstrate the use of (14) in the context of this study, we assume *k* = 5 and two lists of ranked concepts *P*_*k*_^*Z*^ = {2, 3, 1, 6, 8} and ${P}_k^{d_i}=\left\{2,\ 1,\ 4,\ 3,\ 5\right\}$. Again, an integer represents a unique concept and the rank order of the concepts in each list is from left to right. Table 3 gives the calculation of $RB{O}_{Z,{d}_i}$ step by step.

**Table 3:** Step-by-step calculation of $RB{O}_{Z,{d}_i}$ given *P*_*k*_^*Z*^ = {2, 3, 1, 6, 8} and ${P}_k^{d_i}=\left\{2,\ 1,\ 4,\ 3,\ 5\right\}$

Depth	${P}_{depth}^Z\cap {P}_{depth}^{d_i}$	$\frac{\left|{P}_{depth}^Z\cap {P}_{depth}^{d_i}\right|}{depth}$	${\varphi}^{depth-1}\frac{\left|{P}_{depth}^Z\cap {P}_{depth}^{d_i}\right|}{depth}$
1	1	1/1 = 1	(0.9)^0^ × 1 = 1
2	1	1/2 = 0.5	(0.9)^1^ × 0.5 = 0.45
3	2	2/3 = 0.67	(0.9)^2^ × 0.67 = 0.54
4	3	3/4 = 0.75	(0.9)^3^ × 0.75 = 0.55
5	3	3/5 = 0.6	(0.9)^4^ × 0.6 = 0.39
$RB{O}_{Z,{d}_i}$	(1 − 0.9) × (1 + 0.45 + 0.54 + 0.55 + 0.39) = 0.29		

We use (14) to compute the similarity between *P*_*k*_^*Z*^ and ${P}_k^{d_i}$ for each document. All the documents are then re-ranked according to their *RBO* values.

### Mechnism for keeping user-selected documents

Due to content variations of documents and the subjective nature of relevance, some user-selected documents (as relevance feedback) in the current-round search may not be in the top results any more in the next-round search using the above relevance-based search methodology. A mechanism is developed to keep the user-selected documents in the top results of the next-round search.

Let *χ* represent the top documents of the current-round search a user would like to review. Its value can be set by the user. Let *r* represent the user-selected relevant documents among *χ* in the current-round search. Let ξ represent the documents that belong to *r* but not in *χ'* of the next-round search results obtained using the relevance-based search methodology. That is, *ξ* ⊂ *r* and *ξ* ∩ *χ*' = ∅. We use the documents in ξ to replace the last |*ξ*| number of documents that do not belong to *r* in *χ'* of the next-round search results. The mechanism is demonstrated using the following example.

Assume that *χ* = {*d*_1_, ***d***_2_, *d*_3_, ***d***_4_, ***d***_5_, *d*_6_, *d*_7_, *d*_8_, ***d***_9_, *d*_10_}, where the documents in bold are selected by a user as relevance feedback. That is, *r* = {*d*_2_, *d*_4_, *d*_5_, *d*_9_}. The order from left to right represents the ranked order of the documents. Assume that the ranked top documents of the next-round search results are *χ*^'^ = {*d*_2_, *d*_13_, *d*_11_, *d*_7_, *d*_14_, *d*_1_, *d*_10_, *d*_3_, *d*_5_, *d*_12_}. One can find that two documents belong to *r* but are not in *χ'* any more (i.e., *ξ* = {*d*_4_, *d*_9_}). The replacement process starts from the last document in *χ'* and ξ. That is, *d*_12_ is replaced by d_9_. As *d*_5_ϵ*r, d*_5_ is skipped and not replaced. Next, *d*_3_ is replaced by *d*_4_. Hence, the adjusted next-round top documents are *χ*^'^ = {*d*_2_, *d*_13_, *d*_11_, *d*_7_, *d*_14_, *d*_1_, *d*_10_, *d*_4_, *d*_5_, *d*_9_}, which are returned to the user. The mechanism, on the one hand, keeps the user-selected documents in the top list a user is willing to view. On the other hand, it makes sure that the documents at lower rank positions are replaced since the documents ranked higher are more likely to be new relevant documents. Please note that the user can do several rounds of relevance feedback until his/her information needs are satisfied or he/she simply wants to quit.

## Results

### Experiment data

The Genomics data from TREC 2006 Genomics Track [37] were used to test the effectiveness of our proposed relevance feedback system in this study. The track collected 162,259 full-text documents and 28 topics expressed as questions. These topics were classified into four categories of information needs: 1) information describing the role(s) of one or more genes involved in a given disease; 2) information describing the role of a gene in a specific biological process; 3) information describing interactions (e.g., promote, suppress, inhibit, etc.) between two or more genes in the function of an organ or in a disease; and 4) information describing one or more mutations of a given gene and its biological impact. As the 162,259 full-text documents were too much data to perform an exhaustive expert evaluation regarding whether each document was relevant to each topic, the track created a much smaller separate pool for each topic. Each pool included 1000 passages that were ranked high, relative to a particular topic, by the systems from various research groups involved in the track. These pools of passages were judged by experts invited by the track, where passages were extracted from various documents. The degree of relevance between each topic and a passage was classified by the related expert into three categories: “NOT”, “POSSIBLY”, and “DEFINITELY”. A document was considered to be relative to a topic if one or more of its passages were either “POSSIBLY” or “DEFINITELY” relevant to the topic based on the judge of an expert. Since, in many cases, more than one passage belongs to the same document, the number of documents in each pool is less than 1000. Each pool generally contains from 300 to 700 documents. The number of documents relevant to each topic was from 0 to 234.

Note that the documents were provided as html files by the track. We first preprocessed the original html files by removing all the html tags in them and converted them into text files. These text files were then sent to UMLS servers in order to get the MetaMap files that contained the mapped biomedical concepts. In addition, we also did a simple processing of the selected topics by removing the stop words, punctuation, and so further before they were sent to UMLS servers.

### Experiment results

Given the gold standard provided by the TREC 2006 Genomics Track, no documents were found to be relevant to 2 out of the 28 topics. The rest 26 topics were utilized for the initial search in the experiments. We assume that users are willing to review top 10 or 20 results and select all the relevant documents in the top 10 or 20 as relevance feedback for the next-round search. Among the initial search results, it was found that there were one or more relevant documents for 17 out of the 26 topics in the top 10, while two more topics obtained non-zero relevant documents in the top 20. Table 4 presents the number of relevant documents that were in the top 10 and 20 of the initial, 2^nd^-round, and 3^rd^-round search results when *k* is 30. For topic 14 and 18, no relevant documents were found in top 10, while one relevant document was found in top 20 in the initial search. One can see that, in general, relevance feedback does improve the search results even though its effectiveness is varied for different topics. The experiment results also indicate that relevance feedback has higher impact on the 2^nd^-round search than the 3^rd^-round search. Please note that the table only provides the number of relevant documents without showing the specific rank of each relevant document. For some topics, even though the numbers of returned relevant documents are same (either from initial to 2^nd^-round search or from 2^nd^-round to 3^rd^-round), the specific ranks can be different. For example, top 10 results include two relevant documents in both 2^nd^-round and 3^rd^-round search for topic 2. We checked more details of the results and found that the ranks of the two relevant documents in the 2^nd^-round search were “1, 4”, while their ranks in the 3^rd^-round became “1, 2”. In this case, Therefore, the relevance feedback did result in improvement from the 2^nd^-round search to the 3^rd^-round search for topic 3, even though the improvement is moderate.

**Table 4:** Number of relevant documents in top 10 and 20 for each topic in the initial, 2^nd^-round, and 3^rd^-round search (*k* = 30)

Topic ID	Top 10 results			Top 20 results		
	Initial	2^nd^	3^rd^	Initial	2^nd^	3^rd^
1	7	10	10	14	20	20
2	1	2	1	2	4	4
3	4	7	8	8	13	14
4	1	6	6	1	6	7
5	1	2	2	1	2	2
6	5	5	5	12	13	13
7	10	10	10	18	19	19
8	6	6	6	10	11	12
12	1	2	2	2	3	3
13	1	1	1	2	2	2
14	0	N/A	N/A	1	2	2
15	9	10	10	19	20	20
16	3	3	3	6	8	9
17	2	3	3	2	4	4
18	0	N/A	N/A	1	2	2
19	6	9	9	12	19	20
MAP	0.605	0.842	0.866	0.467	0.728	0.731

The mean average precision (MAP) at 10 and 20 for the initial, 2^nd^-round, and 3^rd^-round search results were computed and included in the last row of Table 4. Average precision (AP) is the average of precision values at all ranks where relevant documents are found. MAP for a set of queries is the mean of the average precision scores for each query. It is a standard single-number measure for comparing literature search algorithms. Both MAP@10 and MAP@20 indicate BiomedSearch can significantly improve search performance, especially from initial to 2^nd^ round search.

We investigated how the parameter *k* affected the search results. Since *k* is only used after receiving a user’s relevance feedback in order to form *k*-profiles for the feedback and each document (see Section III.C), it does not affect the initial search results. We checked the MAP@10 and MAP@20 for both 2^nd^-round and 3^rd^-round search when *k* takes different values (Table 5). The results indicate that the performance of the proposed relevance feedback system is relatively poor when *k* is too small or too big. The reason behind this is that, if *k* is too small, some important concepts may not be included in the *k*-profiles, which causes poor performance as the re-ranking is based on those *k*-profiles. Similarly, if *k* is too high, some concepts that are not relevant to the search topic may be included in the *k*-profiles, which also causes poor performance. Table 5 indicates that 20 or 30 represents a proper value for *k*.

**Table 5:** MAP@10 and MAP@20 for 2^nd^-round and 3^rd-^-round search when *k* takes different values

		*k* = 10	*k* = 20	*k* = 30	*k* = 40	*k* = 50
MAP@10	2^nd^	0.807	0.827	0.842	0.813	0.806
	3^rd^	0.816	0.840	0.866	0.824	0.812
MAP@20	2^nd^	0.703	0.708	0.728	0.715	0.703
	3^rd^	0.717	0.719	0.731	0.721	0.716

To get a more in-depth understanding of the effect of *k*, we randomly chose a topic with moderate number of relevant documents and checked the ranks of all these relevant documents when *k* takes different values. Topic 4 had totally eight relevant documents and was randomly chosen for this experiment. As relevance feedback exhibits relatively high impact on the 2^nd^-round search, we provide the ranks of all the eight documents relevant to topic 4 when *k* takes different values in Table 6. Each document ID is the PMID (unique identifier used in PubMed) that was designated by Highwire Press from which all the documents were obtained by the track. One can see that, if *k* is small, the variation of the ranks is more significant. When *k* becomes bigger, the ranks are more consistent. If *k* is too big (e.g., *k* = 50), the performance of the system becomes a little bit worse. Another interesting observation is that, when *k* takes 30, 40 or 50, almost all documents are ranked high except the last one in the table (i.e, the document 15452128). This implies that the *k*-profile for the last document is quite different from those for the other documents. This situation is possible since, in some exceptional conditions, a topic can be discussed in a paper from a different field. Please note that, if one passage extracted from a document is considered to be relevant to a topic by a judge from the 2006 Genomics Track, the whole document will be considered to be relevant.

**Table 6:** Specific ranks of the documents relevant to topic 4 in the 2^nd^-round search when *k* takes different values

Document ID	Ranks of the Documents Relevant to Topic 4				
	*k* = 10	*k* = 20	*k* = 30	*k* = 40	*k* = 50
15003956	1	1	1	1	1
1528178	2	2	2	2	2
9302273	4	5	4	4	4
12867662	6	3	6	6	6
9328480	7	14	7	8	8
9700208	11	10	10	10	11
9516475	96	111	24	24	25
15452128	173	192	174	174	176

In the experiments presented above, a document was considered to be relevant to a topic if at least one of its passages exhibited either “POSSIBLY” or “DEFINITELY” relevance to the topic. As relevance feedback relies on users’ effective selection of relevant documents as feedback, different gold standards may affect the experiment results. We examined the experiment results when the gold standard only included those documents in which at least one passage exhibited “DEFINITELY” relevance. We call these documents highly relevant documents. With this new gold standard, no relevant documents were found for 5 out of the 28 topics. For the rest 23 topics, the initial search failed to find any relevant documents in top 20 for 4 topics. Table 7 presents the number of relevant documents in top 10 and 20 for each of the 19 topics in the initial, 2^nd^-round, and 3^rd^-round search using highly relevant documents as gold standard. The table also includes the MAP@10 and MAP@20 for each round of search. By comparing Table 4 and Table 7, one can see that the performance was improved for all rounds of search if only highly relevant documents were used as gold standard.

**Table 7:** Number of relevant documents in top 10 and 20 for each topic in the initial, 2^nd^-round, and 3^rd^-round search using highly relevant documents as gold standard (*k* = 30)

Topic ID	Top 10 results			Top 20 results		
	Initial	2^nd^	3^rd^	Initial	2^nd^	3^rd^
1	7	10	10	14	20	20
2	1	2	2	2	4	4
3	4	7	8	8	13	14
4	1	6	6	1	6	7
5	1	2	2	1	2	2
6	5	5	5	12	13	13
7	10	10	10	18	19	19
8	6	6	6	10	11	12
12	1	2	2	1	3	3
13	1	1	1	2	2	2
14	0	N/A	N/A	1	2	2
15	9	10	10	19	20	20
16	3	3	3	6	8	9
17	2	3	3	2	4	4
18	0	N/A	N/A	1	2	2
19	6	9	9	12	19	20
MAP	0.605	0.842	0.866	0.467	0.728	0.731

## Discussions

In BiomedSearch, association mining techniques are used to find the top *k* concepts that are statistically associated with a given query from the user-selected document(s) (as relevance feedback). These top *k* concepts include more extensive information about a user’s query intention. From this perspective, association mining functions as query extension. Experiment results indicate that this approach can effectively improve the search performance. We believe that BiomedSearch would be even more useful when a user is not sure about what he/she wants or has difficulty in finding the correct keywords to represent his/her intention.

BiomedSearch supports binary relevance feedback. That is, users only need to indicate whether a document is relevant or not for a query. RefMed [10] proposed by Yu et al. is a multi-level relevance feedback system which requires more accurate information about users’ feedback, but, at the same time, puts more burdens on users. States et al. developed a prototype of an implicit relevance feedback where feedback is inferred from users’ search behaviors without users’ explicit inputs [14]. The effectiveness of this type of system is often user-dependent as different users have different search habits. Our approach is a balance between these two systems. Due to lack of relevant information (e.g. users’ behavioral information), our system is not directly comparable with the two systems using the TREC 2006 Genomics data.

BiomedSearch was tested against the gold standard provided by the TREC 2006 Genomics Track. However, when the gold standard was established, each passage extracted from the documents in each pool was only evaluated by one judge. Hence, the standard is subjective and person-related. The track examined the agreement between judges by randomly selecting a total of six topics for judgement in duplicate. The results indicated that, for one of the six topics, the agreement was very low (with a kappa statistic value of 0.028) since “one judge interpreted relevance to the question very broadly and the other very narrowly [37]”. For the other five topics, the kappa statistic indicated “good” instead of “excellent” inter-rate agreement, with a kappa statistic value of 0.60. This weakness of the gold standard provides another potential explanation about the outliner ((i.e, the document 15452128)) presented in Table 6 since the document might not be actually relevant to the topic if it was judged by other biomedical professionals.

BiomedSearch relies on UMLS’s reliability and its effectiveness in breaking sentences into phrases and mapping them to standard biomedical concepts. Fortunately, UMLS is well maintained and consistently updated by NLM. NLM not only provides a cluster of servers and related software packages and interfaces to support UMLS mapping but also offers lexical and text tools to manage lexical variations and index raw text files. Using these tools to pre-process the text files (e.g., removing genitive, stop words, etc.) can potentially improve the mapping results. This needs further investigation and represents our future work.

## Conclusions

We have developed a UMLS-based relevance feedback system for biomedical literature search. UMLS Metathesaurus was utilized to map text files to standard biomedical concepts. We employed association mining techniques to construct a *k*-profile from a user’s relevance feedback in order to represent the user’s intention for future searches. The profile contains the top *k* concepts that are associated with the user’s query. To find the strength of association between the query and each concept, we proposed a *weighted interest* measure which supports partial matching between the query and each sentence in a document. Preliminary experiment results indicated that BiomedSearch could effectively utilize users’ feedback and improve search performance. We also tested the parameter *k* and found that 20 or 30 seemed to be a proper value.
